# A Practical Treatise on Diseases of the Eye

**Published:** 1831-04-01

**Authors:** 


### A PRACTICAL TREATISE ON THE DISEASES OF THE EYE. By *William Mackenzie*, Lecturer on the Eye in the University of Glasgow, and one of the Surgeons to the Glasgow Eye Infirmary. 8vo. pp. 861. London, 1830.

IT is not because this book is a bad one that we have omitted making it the subject of review ere this. Other matters have occupied our attention, and the substance of other works has been communicated to our readers. It is out of the question to analyse a work consisting of 861 pages, and professing to treat of all the diseases of the eye. The attempt would be preposterous. And yet we are unwilling to forego all the stores of information and utility which such a volume must contain. Ophthalmic surgery has made such rapid advances within the last half century, that nine surgeons out of ten are becoming tolerable oculists; whereas, beneath the old regime, many men of eminence and talent asserted openly and without scruple, that they knew nothing whatever of the matter. Under such circumstances the Medical Journals of the day must lend their helping hand to this department of professional study. Many country practitioners, ill treated and ill paid, can barely afford the small expense which a periodical publication entails, and certainly can afford no more. We purpose from time to time to devote some of our pages to such articles on practical subjects in the department of diseases of the eye, as may prove of service to those readers, who depend on the Medical Journals for the means of keeping pace with the movements of this most active age.

The subject which we shall first select is one which must interest all who have the treatment of any patients at all. It is the OPHTHALMIÆ, or INFLAMMATORY DISEASES OF THE EYE. This is a wider field than many routine practitioners may suspect, or admit. Yet we do not doubt that here, as elsewhere, the theorist, and the subtle wire-drawing nosologist may lay down distinctions without real differences, enjoin diagnoses which are either impossible, or, if possible, useless; and prescribe varieties of treatment, ridiculous in the abstract, and impracticable in the performance. This is the curse of studying diseases too much in detail. A man who dedicates his life to the study of diseases of the eye, or the ear, or the toe, can never rest satisfied without refining. He is galled if that which exclusively occupies himself, be not made so extensive and so complicated as to occupy exclusively all who direct their attention to it. In fine he too frequently becomes a quack and conceals the little that he knows, or spreads it out that it may appear imposing and large, and endeavours to erect a small part of a system or a science, into a science or system by itself. We do not affirm that such is the error of Mr. Mackenzie; we deny that. Yet we think that his volume might be compressed with some advantage, and, if we were disposed to hint a fault or hesitate dislike, we should say that too much pains are taken to comprise every thing within the 900 pages. But enough of this for the present.

#### OF THE OPHTHALMIÆ IN GENERAL.

Inflammation has many stages, and we need not tell our readers now that it has also many characters, determined by the texture in which it occurs. Since the days of Bichat attention has been closely and most successfully directed to the influence of tissue on disease, or, to speak more philosophically, to the nature of the morbid alterations of the tissues.

"The conjunctiva, sclerotica, cornea, iris, chrystalline capsule, and retina, present a series of the modifications of inflammation, to which I have just now referred, sufficiently distinct to convince the most sceptical of the truth of what I have been asserting, and sufficiently striking to rouse the most inattentive to research. The muco-cutaneous conjunctiva secreting a flood of purulent matter, as in the ophthalmia of new-born children—the fibrous sclerotica affected for months with rheumatic inflammation—the transparent fibro-cartilaginous cornea becoming opaque, or being destroyed layer after layer by a penetrating ulcer—the erectile iris losing all power of executing its motions of expansion and contraction—the chrystalline capsule pouring out coagulable lymph from its serous surface, and this lymph forming the medium of morbid adhesions—the nervous retina, too deeply seated to be observed immediately, but in a few hours losing its inconceivably delicate sensibility—these are facts in which are displayed the modifications of inflammatory action and the various consequences of inflammation, fully as distinctly and as strikingly as they are manifested in any other, nay in all the other parts of the body put together." 318.

But the inflammatory diseases of the eye are subjected to the control of other agencies, of other influences natural or artificial. Scrophula, syphilis, gout, mercurialism excite or modify inflammation in its textures. Inflammation too, beginning in one tissue extends to another: from without to within, from within to without. No doubt, many different inflammatory affections of the eye have been lumped together under the convenient term of ophthalmia; but, on the other hand, to describe inflammation as running a defined course in one texture of the eye, and in that alone, is to describe what scarcely exists in nature. Still, for the sake of study, of science, and of beneficial treatment, it is well to be as precise in our notions and descriptions of disease as its nature will permit. The remedies advantageous in inflammation of the sclerotica are injurious in that of the conjunctiva, and so on; yet many have a small stock of nostrums for ophthalmia, which, like Procrustes' bed, must fit all comers, and if it fails they are naturally brought to their wit's ends. Like a celebrated oculist, they spoil their "hatful of eyes," but, unlike that gentleman, they are little the better for the business. The following is the arrangement of ophthalmiæ proposed by Mr. Mackenzie.

"I. CONJUNCTIVITIS.

i. Conjunctivitis Puro-mucosa.

1. Catarrhal.—2. Contagious or Egyptian.—3. Leucorrhœal, or Ophthalmia Neonatorum.—4. Gonorrhœal.

ii. Conjunctivitis Scrofulosa.

1. Phlyctenular.—2. Pustular.

iii. Conjunctivitis Erysipelatosa.

iv. Conjunctivitis Variolosa.

v. Conjunctivitis Morbillosa.

vi. Conjunctivitis Scarlatinosa.

II. SCLEROTITIS.

1. Rheumatic.

III. CORNEITIS.

1. Scrofulous.

IV. IRITIS.

1. Rheumatic.—2. Syphilitic.—3. Scrofulous.—4. Arthritic.

V. CHOROIDITIS.

VI. RETINITIS.

VII. AQUO-CAPSULITIS.

VIII. ANTERO-CHRYSTALLINO-CAPSULITIS.

IX. POSTERO-CHRYSTALLINO-CAPSULITIS.

X. VITREO-CAPSULITIS.

XI. CHRYSTALLINITIS.

Appendix.

1. Traumatic Ophthalmiæ.—2. Compound Ophthalmiæ, as the Catarrho-rheumatic, pustulo-catarrhal, &c.—3. Intermittent Ophthalmiæ." 321.

It would be easy to start objections to this arrangement, the distinctions of the genus conjunctivitis, being particularly arbitrary. But this does not signify much. We pass to a short section on the remedies for the ophthalmiæ.

Several general rules may be laid down on this head. First, discover the cause of the inflammation, if possible, and remove it; secondly, defend the eye from new causes of irritation, for the first may have been local, but the latter may be constitutional. Amongst the various remedies which may be employed for the cure of ophthalmia, we may enumerate, 1st. *Blood-letting.* Venesection in the arm, leeches round the eye, and division of the inflamed conjunctiva are generally employed. Opening the temporal artery, the external jugular vein, or the nasal vein, or cupping from the temples is seldom necessary. The three first cannot, as might readily be imagined, be indifferently substituted for each other.

"I know of no inflammatory disease of the eye which is curable by bleeding alone; and I look on the attempts to cure the contagious or Egyptian ophthalmia by taking away very large quantities of blood, till the inflamed membrane grows pale from depletion, as the veriest of folly; first, because even were this paleness produced, it could be no test of the disease being subdued; secondly, because a degree of blood-letting sufficient to produce even an approach to such an effect, would leave the patient in a state of great and unnecessary debility; and thirdly, because the disease could be cured by a much milder plan of treatment. All the ophthalmiæ require other remedies besides the taking away of blood; and, therefore, while we value this means of cure very highly, we must by no means trust to it alone in any case." 323.

The blood should be taken in a full stream, and its quantity be regulated by the circumstances of the case. Leeches should generally be applied on the temple, forehead, and side of the nose, and not on the loose substance of the lids. The number must vary from one to twenty, or more. One leech to the middle of the upper eye-lid is often very useful. In some chronic cases of inflamed and thickened conjunctiva, one or two, fixed on the internal surface of the lids, will be found useful. Temporal arteriotomy, scarifying and cupping on the temples, produce irritation and preclude the use of other means; besides, the bandaging afterwards is injurious. Scarification of the palpebral conjunctiva is a valuable mean of cure. One or two deep incisions should be made along the whole length or the inner surface of both lids, which ought to be alternately everted and allowed to return to their natural position, which greatly promotes the continued flow of blood. Snipping across individual enlarged vessels may be classed with scarification. Mr. Mackenzie raises a small fold of the conjunctiva with the forceps, and snips it away with the scissors; the vessel is seldom thus divided, but it is exposed, and may easily be raised with a small hook from the surface of the conjunctiva, and divided. The removal of a circular portion of the conjunctiva is almost laid aside, and Mr. Wardrop's mode of evacuating the aqueous tumour has never come into general use.

*Purgatives.* An active purge of calomel and jalap, employed early, is often sufficient of itself to cut short an attack. In the course of these diseases occasional laxatives are always necessary; in many cases, especially in children, nothing but a continued use of purgatives will effect a cure.

*Emetics.* These are extremely beneficial in the treatment of various inflammatory affections of the eye; and in chronic cases they promote the absorption of unhealthy depositions. Diaphoretics are also useful adjuvants.

*Alteratives.* Of these mercury is the chief, and of its powers in controling the internal ophthalmiæ, especially iritis, we need say nothing here.

*Tonics.* The scrophulous ophthalmiæ, and almost all others when chronic, are benefited by tonics, and especially by cinchona. The employment of the sulphate of quinine is an immense improvement in ophthalmic practice. The mineral acids and chalybeates are very serviceable in many ophthalmiæ.

*Narcotics.* Two of the most painful ophthalmiæ are the rheumatic and catarrho-rheumatic. Laudanum rubbed on the forehead and temple does much to relieve the pain, but our author looks on the combination of calomel and opium as almost specific. Opium is occasionally employed directly to the eye in vapour and in fomentation. Hyosciamus, belladonna, and stramonium are of inestimable value, in consequence of their power of dilating the pupil. The extract is most commonly smeared on the brow. If severe inflammation be present in the iris, they have little effect; but if the attack is incipient, or yielding, the pupil is speedily expanded.

*Refrigerants.* In the internal ophthalmiæ they are positively injurious, and in most other cases reaction follows their employment. Incipient inflammation of the external covering of the eye may sometimes be checked by cold lotions, but tepid ones are equally as useful and more safe, on which account Mr. Mackenzie seldom makes use of the former. Nitre is occasionally given internally in some ophthalmiæ.

*Astringents.* The argenti nitras and hydrargyri murias in solution have usurped, in our author's hands, the place of all other astringent lotions and drops. Even the cupri sulphas and lapis divinus may be laid aside, excepting in a few peculiar cases.

*Stimulants and Escharotics.* Such are the argenti nitras, calomel, red precipitate, sub-nitrate of mercury, vinum opii, &c. Most of them are destructive in the internal ophthalmiæ, but it is quite the reverse in conjunctival inflammation. Mr. Mackenzie reprobates a calomel or nitrate of silver ointment, but in another place he modifies this opinion. The red precipitate and sub-nitrate of mercury are only used in the form of salve. The vinum opii may be applied pure or diluted. There is no panacea. Counter-irritants are of much service, especially in the chronic stage of ophthalmiæ.

"Having thus gone over the chief classes of remedies employed in the treatment of the ophthalmiæ, I may mention that much is to be effected also, in the cure of these diseases, by dietetical regulations, using *dietetical* in its original and extended sense, and comprehending under it every particular in the *mode of life*. Thus, attention to cleanliness, by the removal of morbid discharges from the eyes, exclusion from an improper degree of light, exposure to pure air frequently renewed, early going to rest, quiet sleep, repose of body and mind, a properly regulated diet, and regulated exercise; all these, and many similar observances, are in a high degree conducive to recovery, while a neglect of one or more of these rules is often the cause of prolonged and severe attacks of inflammation, in different textures of the eye." 327.

### Conjunctivitis in General.

Like the other portions of the mucous system, the conjunctiva is liable to inflammation of a puro-mucous, blennorrhœal, or catarrhal character; and it is affected with diseases evidently partaking of the nature of cutaneous eruptions. The net-work of large, tortuous, scarlet-coloured vessels, admitting of being readily drawn aside, distinguish sclerotitis from conjunctivitis. To be sure, when the sclerotica is inflamed, the conjunctiva sooner or later participates, and vice versâ; yet the inflammation may begin in one part only, and continue to affect that most severely. Passing over a short section on the puro-mucous conjunctivitis in general, we stop at—

### I.—Catarrhal Ophthalmia.

Three ophthalmiæ are excited, especially in adults, by atmospheric influences; the catarrhal, the rheumatic, and the catarrho-rheumatic. The first is a puro-mucous inflammation of the conjunctiva; the second is an affection of the sclerotica; and in the third both the conjunctiva and sclerotica are attacked.

*Symptoms.* Catarrhal ophthalmia, the most common disease of the adult eye, is almost entirely confined to the conjunctiva and Meibomian follicles. The mucous secretion is increased, and occasionally becomes opaque, thick, and puriform; but in many cases it remains transparent. The Meibomian secretion, also increased in quantity and altered, concretes on the edges of the lids and amongst the eye-lashes, and binds them together during the night. In mild cases the redness is chiefly in the conjunctiva lining the lids. Not unfrequently there are spots of extravasated blood beneath the conjunctiva. In severe cases chemosis may take place to such an extent that the cornea may become infiltrated with pus, burst, and slough. Mr. Mackenzie has been led to attribute the destruction of the cornea, in such severe cases of ophthalmia, partly to the mechanical pressure exercised by the enormously distended conjunctiva of the eye-lids and eye-ball.

*Diagnosis.* The most strikingly characteristic sign of catarrhal ophthalmia is the sensation of roughness in the eye, of sand or dirt having got into it. There is usually little head-ache, and, when any, it is seated across the forehead and felt most in the morning. In the rheumatic ophthalmia one of the most remarkable symptoms is the supra-orbital or circum-orbital pain, severely aggravated during the night.

*Causes.* Atmospheric changes, and especially exposure to cold and wet, are the exciting causes of the disease. An indivivual who has been once attacked is more liable to be affected again. In many instances catarrhal ophthalmia has proved epidemic, amongst a number of persons exposed to the same general exciting causes. It has prevailed at different periods in Italy, England, and France. It is said to be more common in Summer and Autumn. Mr. Mackenzie has no doubt that the disease may be contagious; that is, that the discharge, especially when distinctly puriform, if conveyed by any means from the eye of the patient to those of others, will excite a conjunctivitis still more severe, more distinctly puriform, and more dangerous to the transparent parts of the eye, than was the original ophthalmia. Dr. Guillié took the puriform mucus from the eye-lids of some children affected with puro-mucous conjunctivitis, in the Hôpital des Enfans at Paris, and introduced it under the eye-lids of four blind children. They were amaurotic, but the external surface of their eyes was healthy and entire; in all four a regular puro-mucous conjunctivitis was produced.

*Prognosis.* If neglected, or improperly treated, catarrhal ophthalmia will continue for many weeks, producing constitutional disturbance, and local mischief. The conjunctiva, particularly on the upper lid, becomes sarcomatous and rough, and by its friction brings on a vascular and nebulous state of the cornea, perhaps even white opacity of its upper half.

*Treatment.* Our author is convinced that violent general remedies are absurd, and that a local stimulating plan of treatment may be almost entirely relied on. We should give at the commencement a brisk dose of calomel and jalap, and afterwards occasional doses of neutral salts, determining to the skin by the spiritus mindereri, &c. In severe cases, a blister to the nucha is useful, or blisters may be kept open behind the ears. General bleeding is rarely necessary. Scarification of the palpebral conjunctiva is necessary only in cases in which there is some degree of chemosis, and a distinctly puriform discharge.

Weak solutions of acetate of lead, or of sulphate of zinc find no favour in our author's eyes. The feeling of sand in the eye and the inflammation are best relieved by dropping into the eye a solution of the nitrate of silver, of the strength of two to four grains to the ounce. A large drop is to be applied to the eye once a day by means of a camel's hair pencil. As a collyrium, Mr. Mackenzie employs a solution of one grain of corrosive sublimate in eight ounces of water. This to be applied to the eye thrice a day, milk-warm, upon a rag. In mild cases a few drops may flow in upon the eye; in severe ones, it must be injected over the whole surface of the conjunctiva, and especially into its upper fold, by means of a syringe, so that the whole morbid secretion may be removed, and the diseased membrane touched immediately by the solution. About the size of a hemp-seed of red precipitate ointment, melted on the end of the finger, is to be smeared on the edges of the lids at bed-time. The inside of the lids should be carefully inspected daily, and if there be any tendency to a rough and sarcomatous state of the conjunctiva, it should be alternately scarified or leeched, and touched with the solid sulphate of copper or nitrate of silver. Such is Mr. Mackenzie's treatment, and such he avers to have been almost uniformly successful. Catarrhal ophthalmia may be modified by struma, but of this more hereafter.

### II. CONTAGIOUS OPHTHALMIA.

This disease has attracted great attention. We shall endeavour to be as brief with it as possible. It is essentially the same with the catarrhal ophthalmia, but much more severe, and excited by contagion, perhaps by infection.

*Symptoms.* Their order and severity vary in different individuals suffering at the same time, in the same place, and from the same infection. The disease is said to be milder in women than in men; it is more severe as the age is near to puberty; in scrofulous persons it is always tedious, and more likely to destroy the eye. The disease is much more virulent at one period of its occurrence than another; it was worse at the Military Asylum in 1809, than in 1804. The purely inflammatory stage of the disease is often shorter than thirty hours, never surpasses it, purulent matter being then formed. Sometimes the inflammatory stage is so short and rapid as not to come under the notice of the surgeon. The disease appears to commence soon after the application of the contagious or infectious matter to the conjunctiva, but in many cases secretion of purulent matter has taken place before the patient is aware that he has any inflammation. The *healthy* individuals of any community likely to be infected should be examined daily. The right eye is more frequently and severely affected than the left, commonly both are attacked, at an interval of several days. When the symptoms succeed each other with moderate rapidity they occur in the following order.

"A considerable degree of itching is first felt in the evening, or suddenly there arises in the eye the feeling as if a particle of dust were between the lids and the eyeball. This is succeeded by a sticking together of the lids, principally complained of by the patient on awaking in the morning. The eyelids appear fuller externally than they ought to do. Their internal surface is inflamed, being tumefied and highly vascular; and the semilunar membrane and caruncula lachrymalis considerably enlarged and redder than usual. The swelling of these parts is soft, somewhat elastic, slippery, and easily excited to bleed.

In about twenty-four hours after the first symptoms make their appearance, the mucous discharge from the internal surface of each eyelid is considerable in quantity. It is still thin, but somewhat viscid, and begins to be opaque. It lodges at the inner angle of the eye. On everting the lids, their internal surface is observed to be much more vascular and tumid. There is also epiphora present, especially when the patient exposes his eye to a current of air. He complains of a sensation as if the eye were full of sand, but seems to experience but little uneasiness from the light. Not unfrequently, a considerable discharge of blood takes place from the conjunctiva, after which the swelling of the membrane diminishes for a time. This is sometimes repeated several times before the profuse puriform discharge sets in. It does not appear to arise from the rupture of vessels, but rather to come from the exhalents of the conjunctiva, dilated by red blood, or by a mixture of red blood with the transparent fluid which they usually carry.

The inflammation now extends to the whole internal surface of the eyelids. The secretion from the palpebral conjunctiva is much augmented, and becomes more distinctly puriform, being yellowish and thick. In many cases it is so abundant, that on the patient opening his eyes, the matter instantly flows over the cheeks. It irritates the skin, and even excoriates it. The swelling of the conjunctiva of the lids, and especially of the upper, increases with the discharge; partly from a serous effusion immediately under the membrane, partly from an unnatural and inflammatory development of its vascular structure, partly from a similar enlargement of its mucous cryptæ, and of the Meibomian follicles, giving rise to a sarcomatous appearance of the internal surface of the eye-lids." 339.

The disease may proceed no further, remain stationary for weeks or even months, and then gradually subside. In other cases the inflammation spreads rapidly to the conjunctiva of the eye-ball, small spots from extravasation of blood are sometimes observed, and chemosis is established. Sometimes the chemosis is only partial; but commonly, it gradually spreads from the lids over the surface of the eye towards the cornea, which at last it buries. The chemosis is accompanied by redness and swelling of the skin of the eye-lids, sometimes coming on suddenly and attaining its height in a few hours, at others increasing gradually during several days. This sudden swelling renders the lids quite immoveable, occasioning at first inversion, afterwards eversion, especially of the lower. The sensations amount to little more than a sense of weight and stiffness, with little pain if light be excluded. When the conjunctiva of the eye-ball has become affected, the puriform discharge is greatly increased, but it varies in quantity and quality. It may continue with little change for twelve or fourteen days, the conjunctiva of the eye-ball becoming, in the mean time, sarcomatous, though never to the same extent as the palpebral. At length the disease begins to yield, but not unfrequently the internal surface of the lids remains in a sarcomatous state, and excites or maintains a chronic inflammation of the investing membrane of the cornea. This is a tolerably favourable case.

Sometimes the conjunctiva covering the cornea is thickened and in some degree detached. Superficial ulceration sometimes attacks the cornea giving rise to opaque cicatrices, and opposite the ulcerated part the iris sometimes adheres, or the whole substance of the cornea, and even the internal textures of the eye may be affected. There is then deep-seated pulsative pain in the eye, sometimes in paroxysms, sometimes uninterrupted. This pain is often very violent, and its characters vary in various cases, but it chiefly affects the branches of the fifth pair of nerves. The duration of the paroxysm varies; it appears to come on most frequently from 10 to 12 in the evening. During the pain the secretion of tears is more copious, and the purulent discharge almost uniformly diminished. It is only in the most violent form of the disease that frequent paroxysms of pain are met with. Rupture of the cornea frequently takes place under these circumstances, and staphyloma, with loss of sight almost invariably follow.

"The period at which this happens varies exceedingly in different patients. In some the daily occurrence of these paroxysms has continued for a number of weeks before rupture of the cornea is produced. In others, this is effected under the second or third attack, and gives a temporary relief. I say *temporary*, for even this melancholy event does not afford a termination to the disease, and often scarcely checks its progress. The severe pain is seldom present in both eyes at the same time, and although it occasionally happens that the attacks of pain alternate from the one eye to the other, the rupture of the one is generally produced before the severe pain affects the other. In some cases, where both eyes are destroyed by rupture of the cornea, the patient has no recurrence of the pain for some time after the rupture of the first; while in other cases, the pain almost instantaneously shifts to the other eye. It has been known that while the second eye was suffering rupture of the cornea, the first eye, by cicatrizing, was only becoming liable to the same accident again, and this second rupture of the cornea has been preceded by as much pain as was the first." 343.

Rupture of the cornea generally happens when the disease is at its height; the process is one of sloughing and ulceration. Through the opening, or openings, the still clear lens is sometimes seen lying in its capsule, and the internal parts of the eye appear natural. The iris is pushed forward into the opening or openings, it adheres, and partial or total staphyloma is the result. In certain cases it would seem that rupture of the cornea takes place under one of the violent paroxysms of pain, before it has undergone much disorganization. In such a case, related by Dr. Vetch, the aqueous humour escaped by a division of the cornea nearly as clean as if made by a knife. But Mr. Mackenzie believes that this mode of rupture is very rare. Ulceration of the cornea commencing on its surface is one mode by which it is destroyed; infiltration of matter into its substance, is another, and the most frequent. In many cases the disease does not cease with the bursting of the cornea. The capsule of the lens soon ulcerates and the lens escapes; more or less of the vitreous humour follows; and sometimes almost the whole contents of the eye-balls are evacuated, a small and deformed one being left sunk in the socket.

The external symptoms of the disease and the pain cease at very uncertain periods. The external tumefaction of the lids usually disappears first, and then the chemosis. The lids have still a gaping relaxed appearance, and secrete a little matter. In this state, which may continue for months, there is great liability to relapse, and the patient is capable of infecting others. The opacities of the cornea are sometimes removed with surprising rapidity. The enlarged and indurated state of the mucous follicles of the palpebral conjunctiva, and of the Meibomian follicles, and the vascular and nebulous state of the cornea are, in many cases, the latest symptoms to disappear. Mr. Mackenzie quarrels altogether with the term "granular conjunctiva." He says that the granular prominences in question appear to be chiefly the acini of the Meibomian glands enlarged.

*Constitutional Symptoms.* The early stage of this disease is entirely local, but afterwards fever is established. At last there is always much general uneasiness, and sleep is prevented by the paroxysms of nocturnal pain. Great debility comes on, and in Egypt the disease often ended in diarrhœa or dysentery, and sometimes in hectic.

*Causes.* A prominent point to determine is the contagiousness of the disease, or its power of propagation from one person to another. The return of the Egyptian expedition certainly introduced a severe contagious ophthalmia into this country, which afterwards prevailed extensively in regiments that had never served in Egypt, and which accompanied the British troops to almost every foreign station to which they were sent. Of the contagiousness of the malady we need scarcely furnish instances, after what has been recorded on the subject by Dr. Vetch, Sir Patrick Macgregor, and others. We are really sorry that we have not space for the account of a fearful ophthalmia which prevailed in the slave-ship Rôdeur, and has been described by M. Guillié. Of 160 negroes 39 remained totally blind, 12 lost each one eye, and 14 had specks, more or less considerable, of the cornea. Of the crew of 22 men, 12 lost their sight, amongst them the surgeon; 5 lost each one eye, and amongst them the captain; 4 had considerable specks and adhesions of the iris to the cornea. One man only remained unaffected, to manage the vessel, and he was seized with the ophthalmia, three days after arriving at Guadaloupe.

*Treatment.* If employed early, the treatment recommended for catarrhal ophthalmia may generally check the disease. If it be more advanced, and especially if chemosis be present, blood-letting to syncope, followed within two hours by leeches will be necessary. The leeches ought not to be placed upon the lids, especially if the integuments are swollen and red, as the bites are apt to fester. We think the following remarks too judicious in the present days of venesection, to be passed unnoticed.

"We ought neither to delay the abstraction of blood, if the symptoms are smart, and the case of some days' standing; nor ought we, on the other hand, to indulge in the absurd expectation that profuse blood-letting is to check the disease completely, without the use of local applications. I hold any notions of this kind, which some may have entertained, as crude and irrational, and their practice as perhaps the most destructive which could be followed. By very profuse blood-letting, the patient is too much reduced, and the eye rendered more susceptible of disorganization. We must not for a moment indulge in the fancy that the stream of blood is to be allowed to flow, till the redness of the eye fades under our view, nor are we even to make the cessation of pain in the eye the condition for stopping the bleeding. These effects might not be obtained by abstracting 50 or 60 ounces of blood, whereas the same real benefit will follow in the course of an hour or two, if not more than 20 or 30 be taken, the patient will be less debilitated, and the course of the disease will with greater certainty be abridged.

Bleeding from the arm may with propriety be repeated, if in the course of 24 or 36 hours after the first venesection, the symptoms have not abated, or have increased in severity. Afterwards, also, should there be any signs of a renewal of inflammatory action, more blood is to be taken away. It is chiefly in cases where there is pulsative pain in the eyeball, and circumorbital pain, coming on in nocturnal paroxysms, that repeated general blood-letting is necessary." 356.

Mr. Mackenzie speaks highly of scarification of the conjunctiva; it may be repeated every second or third day. The antiphlogistic regimen, and a dark, well ventilated room are absolutely necessary. Purgatives are useful in all cases, and especially the emeto-purgatives, as tartar emetic with sulphate of magnesia. Diaphoretics are useful as soon as the active inflammation is subdued. Calomel and opium are of the highest benefit, next to copious venesection, in severe cases, attended with nocturnal circumorbital pain. Bark and other tonics are only useful in the chronic stage.

The local treatment is of no mean importance, and paradoxical as it may appear, it should be alternately soothing and stimulating; either plan too exclusively employed will destroy the eye. It is of great importance that the eye should be frequently cleaned, which is best accomplished by syringing into it with some force a tepid solution of one grain of the oxymuriate in eight ounces of water. Our author decidedly condemns sugar of lead and sulphate of zinc injections. We do not share his horror of them. He prefers greatly the solution of the nitrate of silver, of the strength of about four grains to the ounce. If it fail, a solution of six grains of the sulphate of copper in an ounce of water may be substituted for it as an injection. The red precipitate ointment, or the citrine rubbed along the edges of the lids at bed-time, answer best in preventing them from adhering. Blisters on the nucha, or behind the ears, ought always to be employed.

Much relief is sometimes obtained from allowing the steam of hot water with laudanum to rise into the eyes; or from fomenting them with warm decoction of poppy-heads. Rubbing the head with warm laudanum when the circumorbital pain threatens to commence is also highly useful. Evacuation of the aqueous humour has been employed with the view of relieving the severe pain in the eye and head, and preventing bursting of the cornea. Our author conceives that it will seldom be required if the remedies already recommended be had recourse to. When the purulent discharge is gone the vinum opii proves an excellent application to the relaxed conjunctiva.

Preventive measures are of the highest consequence, especially to military surgeons. If troops be sent to countries where this disease prevails, let them guard against the exciting causes of catarrhal ophthalmia. Soldiers on guard or at bivouac should cover their heads well during the night, and in moist or cold situations should avoid currents of air as much as possible. As soon as there is any appearance of puro-mucous ophthalmia in a regiment, the medical officers should institute a daily and minute inspection of every individual belonging to it. Those affected should immediately be separated from the rest, and not be allowed to rejoin their companies till perfectly cured. Excessive crowding of the men together, especially in their dormitories, should be carefully avoided. The affected should be acquainted with the contagious nature of the disease, and warned against the modes in which it is likely to be communicated. Barrack towels should be entirely laid aside. It will be found salutary to parade the men frequently in their respective companies, with separate vessels of water, while an officer sees their faces and eyes carefully washed.

### III. Ophthalmia of New-born Children.

This is a subject of the greatest importance to every practitioner. The disease is common; occasionally, we rejoice to say rarely, severe and shocking in its consequences under scientific treatment; but a thousand times more severe and terrible under bad. It is a puro-mucous inflammation, and has been termed the *ophthalmia neonatorum*, or *purulent ophthalmia of infants*. The sentiments of our author on the origin of the disease are deserving of serious attention.

"We have reason to believe that this disease is, in general, an inoculation of the conjunctiva by leucorrhœal fluid, during parturition; and that, therefore, it may be prevented, in almost all cases, by carefully washing the eyes of the infant with tepid water, as soon as it is removed from the mother. This is too seldom attended to; the child is allowed to open its eyes, the nurse sitting down with it on a low seat before the fire, or in a draught of cold air from the door, and nothing is done to the child for perhaps half an hour or longer. Exposure to the light, to the heat of the fire, or to the cold draught from the door, are all likely enough injurious to excite the eyes of the new-born infant; and, accordingly, some have been led to attribute the purulent ophthalmia which so frequently shows itself about the third day after birth, to these causes. It will, in general, be found, however, that when the child becomes affected with this ophthalmia, the mother has had leucorrhœa before and at parturition, and that the eyes have not been cleaned for some time after birth. To this the ophthalmia seems to be owing, for, like a disease communicated by contagion, it is sudden in its attack, and much more violent than we almost ever see catarrhal ophthalmia, so that it resembles in this respect the Egyptian, or the gonorrhœal inflammation of the conjunctiva. That some of the cases of purulent ophthalmia, in infants, are catarrhal, is by no means unlikely; occasionally they may arise from the application even of gonorrhœal matter from the mother; but by far the greater number, I believe to be the consequences of leucorrhœal inoculation." 361.

*Symptoms.* It is usually on the morning of the third day after birth, that the eyelids of the infant are glued together by concrete purulent matter. If opened a drop of thick white fluid is discharged, and the inside of the lids is found extremely vascular and much swollen. If neglected or mistreated the swelling rapidly increases, the purulent discharge becomes very copious, and the skin of the lids assumes a dark red colour. In this state the eyes may continue for eight days, or even longer; but about the twelfth the cornea is apt to become infiltrated with pus, its texture is speedily destroyed, it gives way by ulceration, first of all exteriorly to the pus effused between its lamellæ, and then through its whole thickness: and this in a small spot only, or over almost its whole extent, so that sometimes we find only a small penetrating ulcer, with the iris pressing through it, and at others the whole cornea gone and the humours protruding. The disease in its early and manageable state is but too frequently neglected by the attendants, and misunderstood by the medical practitioner. Mr. Saunders imagined that the destruction of the cornea is effected by sloughing and not by suppuration and ulceration. Mr. Mackenzie is convinced of the contrary. Onyx, or infiltration of pus between the layers of the cornea, is the uniform harbinger of destruction; the lamellæ exterior to the pus give way by ulceration; and the ulcer spreads and deepens till the cornea is penetrated, often entirely destroyed. The little patients are fretful and uneasy, with white tongue and disturbed bowels. If the disease is neglected, the flesh wastes and the integuments become loose and ill-coloured.

*Prognosis.* "When a child is brought to us with this disease, our first business is carefully to clean and examine the eyes, explaining to the nurse the manner in which she is to remove the purulent discharge from time to time, and stating plainly what is likely to be the result of the morbid changes already present in the corneæ. If these important parts are only free from ulceration, and from purulent infiltration, however violent the inflammation may be and profuse the discharge, our prognosis may be favourable—the sight is safe. If there is superficial ulceration, without onyx, probably a slight speck may remain. If the ulceration is deep, an indelible opacity must be the consequence. If the iris is protruding through a small penetrating ulcer, the pupil will be permanently disfigured, and vision more or less impeded. If the ulcer is directly over the pupil, the probability is that the pupillary edge of the iris will adhere to the cicatrice, and vision be lost until a lateral pupil be formed in after-life by an operation. If there is a considerable onyx, we can promise nothing, for although under proper treatment, the matter may be absorbed, this is by no means a certain result; the purulent exudation may, on the contrary, increase, the cornea burst, and the eye become partially or totally staphylomatous. Whenever the person who brings the child to me announces that the disease has continued for three weeks, I open the lids of the infant with the fearful presentiment that vision is lost, and but too often I find one or both of the corneæ gone, and the iris and humours protruding. In this case, it is our painful duty to say that there is no hope of sight." 363.

*Treatment.* The purulent discharge must be removed three or four times in the course of the day with a sponge and the tepid corrosive sublimate collyrium. Evert the lids, and wipe them clean with the sponge; and, if there is a tendency to continued eversion of the upper lid, push the swollen conjunctiva into its place, and bring down the edge of the lid. Besides the sublimate collyrium, the solution of the argent. nit. or cupri sulph, (four grains of the former, or six of the latter to the oz. of water) should be applied once, or at most twice a-day, with a large camel-hair pencil, to the whole surface of the inflamed conjunctiva. To prevent adhesion of the eyelids at night, the red precipitate ointment should be applied along their edges at bed-time. If employed within the first two or three days, these measures are usually successful. If the case has been neglected for eight or ten days scarification of the conjunctiva, or the application of a leech to the external surface of the upper eyelid, is necessary. The latter may be first employed, and if not effectual, scarification may be employed next day. Blisters to the back of the head, or behind the ears, are of great service. Cantharides ointment spread on a bit of candle-wick, and laid between the head and the external ear, is a convenient mode of blistering. An occasional dose of castor oil is useful. Recovery from this disease is often very tedious, but when the symptoms have become stationary, Mr. M. has found from a quarter to half a grain of calomel daily very beneficial. Mr. Saunders recommended extr. cinchon. in threatened disorganization of the cornea, but our author prefers the sulphate of quina. The relaxed conjunctiva, remaining after the purulent discharge, may be advantageously touched once a-day with the vinum opii.

The succeeding section, which treats of Gonorrhœal Ophthalmia, we shall omit, having lately descanted so fully on the subject in our analysis of Mr. Lawrence's work. We pass to another variety of ophthalmia.

### IV. Scrophulous Ophthalmia.

This is a very common disease, with highly characteristic symptoms, constituting 90 out of the 100 cases of inflammation of the eyes in young subjects. It is most prevalent from the time of weaning till about 8 years of age. Sometimes only one eye is attacked, sometimes both, and not unfrequently the disease passes from the one eye to the other. When both are inflamed at once, one is generally much worse than the other.

*Symptoms.* Redness at first is very slight. Sometimes a few scattered vessels are seen coursing through the conjunctiva towards the cornea, sometimes there is merely intolerance of light. Mostly three or four enlarged vessels are seen running towards the cornea, or over its edge towards its centre; they are evidently superficial. Not unfrequently they form a considerable fasciculus, and at the end of this a pimple is very likely to appear, if it be not already present. Sometimes the redness is pretty general over the conjunctiva from the first; as the disease advances it becomes increased, and the sclerotica appears somewhat inflamed.

This ophthalmia is an eruptive disease, one or more minute phlyctenulæ, or minute pustules, arising at the apex of each of the bundles of blood-vessels. Often a single minute elevated point, of opaque white colour, near the centre of the cornea, is all that is seen; at other times, numerous pustules or phlyctenulæ are scattered over different parts of the conjunctiva, some on the cornea, and others over the sclerotica. The edge of the cornea is their frequent situation; the smallest are upon the cornea.

"Beer has particularly mentioned phlyctenulæ as distinguished from pustules in this eruptive ophthalmia. We unquestionably meet with pimples of different sizes in this disease. Some patients have them all small like what are termed phlyctenulæ, and others have them all large like pustules. The former contain a smaller quantity of fluid, and that thin and colourless. The fluid contained in the latter is greater in quantity and more like pus. I have not been able to decide whether there is any specific difference between the phlyctenular and the pustular cases. I have frequently observed that the pustular cases are not, in general, attended with so much intolerance of light. The cases in which children lie for weeks and months with their eyes shut, are phlyctenular. The pustular variety certainly does not differ from the phlyctenular merely in the inflammatory action being more severe in the former; for we meet with cases of very large pustules, in which the inflammation and pain are moderate, compared to what attend some cases of phlyctenula. The ulcer which succeeds to phlyctenula is sometimes superficial, but at other times it grows deep, and penetrates into the substance, or even through the cornea, so that no distinction can be grounded on the kind of ulcer which follows the bursting of these pimples." 383.

The phlyctenulæ and pustules may be absorbed; if on the cornea, they leave a little albugo round them, which is generally absorbed in the course of time. Sometimes the albugo begins to spread over the cornea irregularly, pretty large red vessels running into it, and additional lymph being supplied, so as to form a "*vascular speck*," which is very tedious and troublesome. Often the pimples burst and become small ulcers, which are very annoying, and, on the cornea, are apt to obscure vision permanently by their opaque cicatrices. If the ulcer be on the cornea, and allowed to eat through it, a little fistulous opening of the cornea is produced, the aqueous humour is discharged, and a small portion of the iris protruding looks like the head of a fly, and is termed *myocephalon*. If several pustules form on the cornea at the same time, they sometimes unite with each other before they burst, purulent matter is infiltrated between the layers of the cornea, and thus a kind onyx is formed. If the whole thickness of the cornea has been destroyed by ulceration, excepting its inner lining membrane, this is protruded through the opening in the form of a small vesicle; and this is termed hernia corneæ. At last this vesicular prominence gives way, and the aqueous humour is discharged. When there has been extensive prolapsus of the iris through an ulcer of the cornea, the pseudo-cornea formed over the iris is sometimes unable to withstand the presence of the aqueous humour, and is pressed forwards so as to form a partial staphyloma.

Intolerance of light is a very marked, general, and distressing symptom of scrofulous ophthalmia. It is always most severe in the morning, sometimes remitting so much in the afternoon as to allow the patient to open his eyes, and see to a very considerable degree for some hours. On exposure to light, the intolerance is accompanied with strong spasmodic action of the orbicularis palpebrarum. These symptoms are not most severe in the most severe cases, far from it. It is frequently not near so intense in cases where there are large pustules on the conjunctiva covering the sclerotica, as where the eruption is more of the phlyctenular sort, or even where there is yet no pimple formed. There is always epiphora, or profuse flow of tears, often violent fits of sneezing. The irritation of the tears occasions a pustular eruption on the face, which is sometimes rendered exceedingly swoln, red, and painful. Pain does not appear to be a general attendant on the disease; and the intolerance of light appears rather to be produced by a sensation of intolerable glare and dazzling on exposure, than by absolute pain of an inflammatory character. But pain during the night is not uncommon, the child often awaking screaming with it. There is usually a great degree of itchiness, making the patient rub the eyelids very much, and a feeling of sand in the eye, but less than in the catarrhal ophthalmia. Mr. Mackenzie accounts for the epiphora, intolerance of light, and spasmodic action of the orbicularis by the anatomical fact, that the lachrymal nerve, after supplying the lachrymal gland, goes to the conjunctiva and orbicularis palpebrarum.

Sometimes there are other local symptoms. The iris may be inflamed, though this is more usual in connexion with corneitis. Inflammation of the choroid or retina is still more rare; ophthalmia tarsi is very frequent. Other scrofulous symptoms are almost universally present with scrofulous ophthalmia, and occasionally alternate with it. Our author has seen this the case with scrofulous swelling of the knee. A porriginous eruption is frequently met with on the scalp, and an impetiginous eruption over the body, especially in those who live much on milk. The belly is generally hard and tumid, the bowels disordered, the whole frame relaxed and weak.

*Remote or predisposing Causes.* Of these, the scrofulous habit of body must be considered the chief. It were idle to enter here on the discussion respecting the nature or absolute existence of scrofula. All practical men know well what is meant, and that for the present is sufficient. Mr. Wardrop has described what he calls the *exanthematous ophthalmia*, but our author considers it nothing more than the scrofulous ophthalmia, and hits Mr. Wardrop rather hard on the occasion. Mr. Christian, of Liverpool, has described a *porriginous* ophthalmia, produced, as he says, by porriginous matter, carried on the fingers of the child from the ears or head to the eyes. The hint may be worth attention. Improper diet, want of air and exercise, and insufficient clothing are, of course, very frequent remote causes of the disease in large towns and among the lower classes. Our variable climate is also very operative; new attacks are most prevalent during North-easterly winds.

*Exciting Causes.* Measles, small-pox, scarlet fever, themselves affect the eyes, leave them tender, and make them liable to this ophthalmia. Catarrhal ophthalmia is apt to degenerate, in scrofulous children, into the scrofulous. Excessive use of the eyes, especially by candle-light, teething, local injuries, are all causes of this disease.

*Prognosis.* This must be very guarded. No disease is so liable to relapse, and of this the parents should be made aware. When there are ulcers on the cornea, specks must necessarily follow. These will be more or less obstinate, according to the depth of the previous ulceration, and will impede vision in proportion to their extent. Perforating ulcer, followed by protrusion of the iris, leaves almost uniformly a dense leucoma, with deformed pupil.

*Treatment.* Under any treatment, scrofulous ophthalmia is frequently rebellious, and, till we discover a more certain and successful method of treating scrofula itself, it is likely to remain so.

"Is it incurable then? Are we to do nothing for it; but shake our heads, and leave the eyes to be destroyed? Not at all. Much may be done to relieve this disease. Although it is very difficult to cure it thoroughly, especially when the patient continues exposed to the influence of the same causes which originally produced it, yet it is rare indeed that medical treatment does not moderate the symptoms, and avert those changes in the transparent front of the eye, which in neglected cases are so often the causes of loss of sight. But when the practitioner does meet with cases, as sometimes he must do, which receive no benefit for weeks and months, but perhaps rather get worse, notwithstanding all that is done for them, he must not blame himself too much, but reflect on the intractable diathesis with which, in such cases, he is called to contend, and which he cannot change, and but too often can scarcely in the smallest degree ameliorate." 391.

We must always attend to the general health in the treatment of this ophthalmia. General blood-letting is hardly ever required, local not often. When inflammatory action runs unusually high, or is suddenly augmented by the formation of pimples or ulcers on the cornea, leeches to the eye-lids or temple are proper. If the affection is yet recent leeches should be frequently employed, whilst the redness of the conjunctiva is considerable, and the intolerance of light acute. Tartar emetic will often supersede the necessity for depletion, and by depletion alone no case of this disease can be cured, but much mischief may be perpetrated. Our author speaks most highly of tartar emetic. He usually commences his treatment with an emetic, either of ipecacuan or antimony, and with uniform good effects. If there is much quickness of pulse, he usually employs a course of nauseants, or emeto-cathartics. To an adult he gives the combination of emetic tartar with sulphate of magnesia, first as an emetic, and afterwards repeated according to circumstances. In chronic cases the antimony may be substituted at longer intervals and in pills, each containing from a quarter to half a grain. To children, from the 12th to the 6th of a grain may be given, according to the age of the child, thrice daily ; in conjunction with salts, or in solution by itself, or in powder with a little sugar. Purgatives are always of great service. In recent cases a purge of calomel, with jalap, rhubarb, or scammony, will often "put out" the attack. It should be repeated at intervals of two, three, or more days, according to circumstances, but though highly beneficial, care must be taken not to push the debilitating action too far. The purgative plan is particularly useful when there is an impetiginous eruption over the body. Tonics are, of course, strikingly beneficial. Our author prefers to all the sulphate of quina in doses of half a grain to very young children, two grains to adults, rubbed up with a little sugar and given thrice a day. This medicine has often acted like a charm, in Mr. Mackenzie's hands. It may be given after an emetic and a purge, or the subdual of inflammatory excitement by tartar emetic. Next to quina stand the chalybeates, of which Mr. M. prefers the precipitated carbonate of iron, and the tartrate of potass and iron. They are most effectual in the pustular form. Rhubarb and the super-carbonate of soda are very serviceable, and the mineral acids are useful.

When the acute symptoms have subsided the cold-bath is very efficient; at an earlier stage, the warm should be frequently employed. Tonics should be continued after all inflammation has ceased, to prevent relapse. Change of air is an excellent tonic. A dry, warm, in-land air is preferable to the sea-coast, on account of the glare from the sea. Our author reprobates, as well he may, the indiscriminate use of calomel in this and in other diseases at the present day. After local blood-letting with evacuants, calomel and opium are sometimes extremely advantageous, and it may even be pushed so far as to affect the mouth. Diaphoresis is of much importance. The tepid bath every second or third day, followed in adults by the flesh-brush. Dover's powder at bed-time is sometimes very useful, and tartar emetic is so likewise. The diet should be low during the acute stage, rather generous in the chronic. The head should be raised as much as possible, and the child ought not to be allowed to lie burying its face in the pillow.

*Local Remedies.* A broad green shade should be worn over the forehead, but the patient may go abroad in fine weather. A handkerchief bound over the eye is decidedly injurious, heating it and adding to the intolerance of light. In recent and slight attacks evaporating and slightly astringent lotions, applied tepid or cold according to the feelings of the patient, are very serviceable. A decoction of poppy heads with a little alkohol, a weak solution of the acetate of ammonia, a little rose water, or a solution of one grain of the oxymuriate in eight ounces of water, answer the purpose. Cold water generally gives relief, but the subsequent re-action is injurious, and the same may be said of alum, curd, &c. When the symptoms are at all severe, fomentations afford relief. Exposure of the eyes to the vapour of laudanum or of camphor, raised by means of a cupful of hot water is of service. Warm bread poultices, without milk, are useful during the night. Scarification is very valuable in chronic cases. In the vascular speck, division of the fasciculus of vessels running over the sclerotica to the albugo cannot be dispensed with. Blistering is very useful, the intolerance of light being often suddenly relieved by it. The discharge should generally be kept up by some stimulating dressing, or a quick succession of them ought to be applied. Our author prefers blistering to friction with tartar emetic ointment. Issues both in the neck and arm are serviceable.

*Stimulants* are decidedly useful, when well chosen, carefully used, and properly timed; if before the acute inflammatory stage has subsided they do harm. The solution of the argenti nitras and the red precipitate salve are the best, and next to them is the vinum opii. Our author generally uses four grains of the argenti nit. to the ounce of water, and whenever ulceration is present on the cornea recourse should be had to it. A stronger solution may be applied directly to the surface of the ulcer, without permitting the solution to spread over the rest of the eyes. When an ulcer threatens to penetrate deep into the substance of the cornea, or has already perforated the anterior chamber, with or without prolapsus of the iris, the ulcer, or the myocephalon should be touched every second or third day with a pencil of lunar caustic, filed to a sharp point. In central ulcer of the cornea, and even when the edge of the pupil is involved, it is highly advisable to apply the extract of belladonna. In perforating ulcer near the edge of the cornea, Mr. Mackenzie is inclined to refrain from it, for the iris cannot be sufficiently removed from the centre of the cornea, and its palsy favours, rather than prevents prolapsus. Scrofulous ophthalmia is exceedingly apt to recur, and therefore the eyes should be inspected from time to time by the medical attendant.

### V. ERYSIPELATOUS OPHTHALMIA.

This has been described by Beer; it appears to be a rare disease.

*Symptoms.* "It commences with a slight feeling of tension in the eye, and parts immediately surrounding it. The conjunctiva becomes of a pale red colour; and rises in soft, yellowish-red vesicles round the cornea. These take a different form from every motion of the eye-lids, and are sometimes so large as to project from between their edges. On strained or rapid motion of the eye-ball, or eyelids, the patient feels a pricking pain in the eye. When the eyelids are a little open, the vesicles give the patient the appearance of one who is weeping, and we expect that at every moment the tears will drop from his eye; but on a nearer inspection, and on pulling down the lower eyelid, we discover the cause of the mistake, into which we are the more ready to fall, as during this inflammation there frequently is a discharge of tears, especially on sudden changes of temperature. The eye is somewhat impatient of light. No other diseased appearances are observed in the eye itself; but the eyelids seem also to be more or less affected with erysipelatous inflammation. At the end of the acute stage, the pain of the whole eye is increased, still exciting in the mind of the patient the comparison of pressing or stretching, especially on moving the eye or eyelids.

As the disease continues, the redness of the conjunctiva increases. It becomes indeed so generally red, that we discover no longer a mere net-work of blood-vessels, but a general, yet pale, and sometimes livid redness. Yet this pale red colour is not uniform. It is contrasted with spots of different sizes, of a bright red colour, which arise from extravasation of blood into the cellular substance between the conjunctiva and sclerotica. The vesicles become more considerable, and project still more from between the half-opened eyelids. The spaces between the vesicles are covered with a thin white mucus, which is secreted in unnatural quantity by the conjunctiva and Meibomian glands. The discharge of tears is also increased. During the night the eyelids are glued slightly together, so that it is with some difficulty that the patient opens them in the morning; when they are opened the cornea appears somewhat dim; but when the eye has been carefully cleared, we see that the apparent dimness of the cornea arises entirely from the mucus collected on its surface.

As the disease begins to subside, the secretion of mucus returns to its natural quantity, the redness of the conjunctiva gradually disappears, and those portions of that membrane which had been elevated in vesicles, re-approach and re-attach themselves to the tunica albuginea and sclerotica. The discharge of tears ceases to be so frequent and so abundant. Those spots which arose from the extravasation of blood are the last symptoms to disappear. They become of a yellowish red colour. There continues, even for a long time, such a diminution of the connexion between the conjunctiva and sclerotica at these places, that the conjunctiva falls into wrinkles whenever the eyeball is moved. It is long before it recovers completely its natural pliancy and pellucidness." 402.

The disease arises from sudden atmospheric changes, slight blows, stings of insects, &c. A purgative, opening the vesicles with the point of a lancet, and gentle diaphoretics are usually sufficient to effect a cure. We have never seen this affection occur idiopathically, but the conjunctiva is not unfrequently affected secondarily to erysipelas of the face. We have frequently witnessed effusion of serum in such cases between the conjunctiva and sclerotica. No particular treatment was required.

### VI. Variolous Ophthalmia.

Variola was formerly by far the most frequent cause of partial and total staphyloma. Inoculation and vaccination have rendered its ravages more rare.

"In most cases of small-pox, pustules form on the external surface, and on the margins of the eyelids. When they are numerous, as in confluent small-pox, they cause such swelling of the lids as completely to close the eyes. As the disease proceeds, matter is discharged partly from the Meibomian follicles, partly from the variolous pustules, the eyelids are glued together so that the eyes cannot be opened for days, and merely from this state, without any pustules being formed on the conjunctiva, the eyes are irritated and painful. At last, as the disease subsides, the swelling of the lids falls so that they are again opened, and the eyes may be found uninjured. It is in this way that the vulgar talk of persons being blind in small-pox for so many days, and then perfectly recovering their sight. But although the cornea has not suffered in these cases, the eyelids and the lachrymal apparatus are often left in an injured state; and not unfrequently small-pox proves the exciting cause of strumous affections of the eyes and eyelids, which may continue troublesome for years. The small-pox pustules on the lids are apt to destroy the eyelashes, to leave red marks and scars, render the edges irregular, and liable to inflammation and excoriation from slight causes, and to produce ophthalmia tarsi, and very frequently trichiasis and distichiasis. Chronic blenorrhœa of the lachrymal sac, and pustular conjunctivitis, are also frequent sequelæ of small-pox." 403.

To moderate the eruption on the face and its effects, it has been recommended to cover the face with a cloth spread with cerate, and foment it from time to time with chamomile decoction; it can do no harm. When the pustules on the eyelids are fully matured we may evacuate them with a needle; we should carefully remove the crusts which form after the pustules burst, having first softened them with some mild ointment. The lids should be often bathed with tepid milk and water, and rags dipped in the same should be laid over them.

In every case of small-pox there is some redness of the conjunctiva, but unfortunately pustules are more apt to form upon the cornea, and being there to prove destructive. When it bursts the ulcer too often deepens and spreads, the cornea is penetrated, the iris becomes adherent, or partial or general staphyloma of the cornea ensues. In bad cases almost the whole cornea is destroyed by infiltration of matter and ulceration. During the suppurative stage of small-pox it is difficult to say what mischief is going on in the eye under the closed and swollen lids. If there be pain in the ball, with dryness, stiffness, and a sensation of sand in the eye; if the uneasiness be much increased by attempts to move the eye or exposure to light even through the lid; if there be frequent discharge of hot tears, then probably there is acute variolous conjunctivitis, perhaps pustules on the cornea. If the eye is easy and only closed from the state of the lids there is probably no danger.

But Mr. Mackenzie has seen both pustule of the cornea and onyx produced after the general eruption was completely gone. This *secondary variolous ophthalmia* sometimes occurs as late as five or six weeks after recovery from the primary disease; it is not so severe as the primary, but still dangerous to vision. A dull whitish point with surrounding haziness is observed on the cornea; the whiteness extends, perhaps to the 12th of an inch in diameter, and then the part becomes yellow; two or more such points render the whole cornea nebulous, or one large pustule may do so. An onyx may appear at the same time at the lower edge of the cornea. The sclerotica is reddened; there are pain and epiphora on exposure to light. The cornea is seldom destroyed; the matter of the onyx or pustules is sometimes absorbed. In other cases ulceration takes place, and a permanent leucoma remains. The surrounding corneal haziness is gradually dispersed, and vision is impaired according to the situation and size of the leucoma. Vision may often be restored by an artificial pupil, and this operation is frequently applicable to the partial staphyloma.

*Treatment.* The best general treatment of small-pox must be followed. The eyes should be frequently bathed with tepid water or poppy decoction, and the edges of the lids smeared with cold cream. When the lids are too much swollen to allow of any application to the conjunctiva, leeches may be advantageously applied behind the ears or on the temples, and followed, if necessary, by blisters. About the 8th or 9th day of the eruption free purging is useful, and as the lids now begin to be opened a weak solution of the argenti nitras, or diluted vinum opii may be used as an injection. In the secondary ophthalmia, our author has found emetic tartar, given so as to vomit and purge freely, abate the inflammation, and promote the absorption of the pustules and onyx. Leeches and blisters are also useful. When the acuteness of the inflammation is abated a course of sulphate of quina is of much service; and undiluted vinum opii employed once a day is the best local application. Belladonna should be smeared on the brow, in order to keep the pupil dilated.

### VII. Morbillous and Scarlatinous Ophthalmia.

A certain degree of conjunctivitis always attends measles and scarlet fever, but it is not usually severe. Occasionally, we have phlyctenulæ, onyx, and ulcers of the cornea, particularly when the patient is scrofulous. By the dregs of the measles or scarlet fever affecting the eyes we may generally understand, that a scrofulous diathesis has been called into action, and that ophthalmia tarsi, or phlyctenular conjunctivitis has been the result. In measles there is catarrhal inflammation of the Schneiderian membrane, and occasionally the attending conjunctivitis is rather blennorrhœal than eruptive. Our author has seen cases in which the eye had been destroyed by severe puro-mucous ophthalmia, excited by measles. In some rare cases of scarlatinous ophthalmia, the iris and capsule of the lens become affected. Our author operated on a boy, in whom specks of the anterior hemisphere of the capsule were brought on in this way.

*Treatment.* It is seldom required to be active. The eyes should be guarded from strong light, bathed occasionally with tepid water, and the bowels should be kept freely open. If the symptoms are unusually severe, leeches to the temples, and blisters behind the ears, or to the nape of the neck. The argenti nitras solution is highly useful, whether the ophthalmia be eruptive or puro-mucous. Sulphate of quina internally is highly beneficial.

The length to which this article has already extended would render it advisable to conclude here. But only the rheumatic and the catarrho-rheumatic ophthalmiæ remain to be considered, previous to entering on the subject of iritis, and inflammation of the deeper structures of the eye. We are therefore unwilling to leave the present paper thus unfinished, and abandon the consideration of the conjunctival inflammations, without adverting to those rheumatic affections of the sclerotica and conjunctiva, which claim such near relationship to them. We pray the indulgence of our readers, and shall endeavour to be as brief as perspicuity will permit.

### VIII. RHEUMATIC OPHTHALMIA.

This differs in the following particulars from the catarrhal ophthalmia, although both have their origin in atmospheric influences. It has its seat in the albuginea and sclerotica, and extends occasionally to the iris—the chief redness is radiated, or zonular, and seated under the conjunctiva—attacking the fibrous membranes, it is unattended by any morbid secretion from the surface of the eye—the pain is pulsative and deep-seated, not so much in the eye as in the brow, temple, cheek, and side of the nose, and it is severely aggravated from sun-rise to sun-set.

By "rheumatic ophthalmia" Mr. Mackenzie does not wish to imply any rheumatic diathesis, but simply inflammation of the fibrous tissue of the eye, and of the surrounding parts resembling it, excited by exposure to cold. He has always seen it primary, and never metastatic. It resembles rheumatism, but has not been generally recognized as rheumatic. It is comparatively a rare disease. When both eyes are attacked, one is always more severely inflamed than the other.

*Symptoms.* The fasciculi of sclerotic vessels are of bright red colour, and advance in radii towards the edge, sometimes even a little over the edge of the cornea. The conjunctivitis is slight, there is usually no tendency to chemosis, nor do the eyelids take part in the disease. There is uniformly dimness of vision, depending on haziness of the cornea and pupil, attended by slight contraction of the latter and sluggishness of the iris. The latter becomes even slightly discoloured, greenish for instance, if naturally blue, and coagulable lymph may even be effused within the pupil. But severe iritis rarely attends this rheumatic sclerotitis. Mr. Mackenzie has never seen any form of suppuration or ulceration in the disease. The access of light is not generally distressing; the eye feels hot and dry in the early period of the disease, but after a time, especially if the symptoms are abated by blood-letting, there is considerable epiphora. The pain is at first of a stinging kind, and extends from the eyeball to the orbit and neighbouring parts of the head, which feel hot to the hand.

"The pain is strikingly augmented by warmth. It often affects the forehead, the cheek-bone, and the teeth ; extending sometimes even to the lower jaw. Occasionally, it is precisely confined to one half of the head. In some instances, it is severe on the side, or even in the cavity of the nose, or in the ear. But, above all, the eyebrow is its chief seat, and next to it the temple and the cheek. It is not unfrequently the acute pulsatory pain of phlegmon, especially when felt chiefly in the eyeball ; in other cases, and particularly around the orbit, it consists rather in an agonizing kind of feeling, which distresses and wearies out the patience of the person affected. It never ceases entirely, so long as the disease continues ; but it varies much in degree, coming on with severity about four, six, or eight o'clock in the evening, continuing during the night, becoming most severe about midnight, and abating towards five or six in the morning ; till then totally preventing sleep, and occasioning great distress. The patient never fails, in the history he gives of his case, to insist on the nocturnal pain, and with his finger to point out its circumorbital seat. It is much more in the forehead, temple, cheek, and side of the nose, than in the eye. It is reasonable to conclude that in this disease the periosteum in and round the orbit, and the fascia of the temporal muscle, (structures similar to the sclerotica), are also affected with rheumatism ; but the chief seats of the pain are, in all probability, the branches of the fifth pair of nerves distributed to the face, and we may fairly attribute a considerable portion of the pain to the sympathy which these nerves have with those belonging to the eyeball." 409.

A considerable degree of fever attends the disease, increasing with the nocturnal paroxysms of pain. In some the attack is slight, and soon goes off, without permanently injuring the eye. In others it is extremely severe, and if misunderstood, may speedily destroy vision. Not unfrequently the disease becomes chronic, without being very severe.

*Exciting Causes.* The most frequent cause is exposure of the eye to a continued blast of cold air, while the head and face are in a state of perspiration. Of such a nature is travelling during the night, with one side of the head close to the broken window of a carriage, and so forth. It does not seem more apt to occur at one season of the year than another. It is more prevalent when the wind is cold and north-easterly, and attacks people of middle age more than either the young or the old. Probably the causes which excite it, would give rise to strumous ophthalmia in the young, to catarrho-rheumatic ophthalmia in the old. Persons who have once suffered from it are disposed to suffer again.

*Treatment.* Bleeding from the arm is necessary in almost all cases of rheumatic ophthalmia. On this point our author's experience is totally at variance with that of Mr. Wardrop, who maintains the contrary opinion. After the bleeding, Mr. Mackenzie is in the habit of applying next day a dozen leeches round the eye, first repeating the venesection if the pulse be strong and full, and the circumorbital pain not relieved. Calomel and opium are highly useful. Two grains of the former and one of the latter should be given every evening till the gums begin to be affected, when the calomel may be omitted, and ten grains of Dover's powder substituted for the opium. Here again our author's experience differs from that of Mr. Wardrop. An hour before the nocturnal paroxysm of pain is expected, warm laudanum, or equal parts of laudanum and cantharides, rubbed upon the forehead and temple, may lessen or prevent the pain. Blisters repeatedly applied behind the ear and temple, but especially to the nucha, are of service. During the whole course of the disease the pupil should be kept under the influence of belladonna. A laxative glyster every morning, or a small dose of Epsom salts may be exhibited. A stronger purge will carry off the calomel and opium. The warm pediluvium at bed-time, with warm diluent drinks towards evening, will generally excite sufficient diaphoresis. In the chronic stage and during convalescence small doses of sulphate of quina, or of the mineral acids will be advantageous. In old mistreated cases, from eight to twelve drops of Fowler's solution sometimes succeeds well. Local applications are of little service. The lunar caustic solution, and things of that class, so beneficial in the other ophthalmiæ, are highly injurious in this. But when little more than a lingering redness, with weakness of the eye, remains, the vinum opii diluted may be dropped upon the eye twice or thrice, or the pure vinum opii once daily. When the plan now explained has been adopted with necessary vigour, Mr. Mackenzie has never seen any permanent sequelæ left by the disease. So much for the rheumatic ophthalmia.

### IX. Catarrho-Rheumatic Ophthalmia.

This is one of the most common, and one of the most severe and dangerous ophthalmiæ. In old persons it often occasions entire loss of sight, often permanently diminished vision.

*Symptoms.* They are, of course, more complicated than unmixed conjunctivitis or sclerotitis. There is the feeling of roughness or sand between the eyelids and eyeball, the secretion of puriform mucus and puriform Meibomian fluid; and there is the nocturnal paroxysm of racking circum-orbital pain. Sometimes the conjunctivitis is severe, the sclerotitis slight, more frequently the contrary. The conjunctiva and sclerotica are simultaneously attacked, in consequence of the influence of one and the same cause. The redness is evidently both conjunctival and sclerotic; under the moveable net-work of the conjunctiva we perceive the immoveable zonular inflammation of the sclerotica. Chemosis is by no means uncommon, and hides from view the sclerotic redness. The conjunctival discharge is never profuse, and seldom opaque, being generally a mere increase of mucus, rendering the lids unusually moist and slippery. The eyelids adhere together in the morning from the inspissated Meibomian secretion, and they are often externally red and swollen. There is considerable intolerance of light and epiphora, especially where the structure of the cornea is affected. The conjunctival pain is felt most in the morning, or when the eyelids are moved; is referred to the surface of the eye, and sometimes to the forehead. The sclerotic pain is nocturnal, as in rheumatic ophthalmia, and circum-orbital.

The cornea is extremely liable to ulceration, and to effusion of pus between its lamellæ, or onyx. If the disease has been neglected for eight or ten days, one or the other is constantly met with.

"The ulcer is peculiar. It spreads over the surface, rarely penetrating deeply into the substance of the cornea. It generally cicatrises without leaving any opaque speck, the cornea remaining merely irregular, as if part of it had been hacked off with the lancet; and of course vision, from imperfect refraction, is indistinct. Professor Beer and Mr. Wardrop have described this kind of ulcer as attendant on pure rheumatic ophthalmia, but I have never seen it except in catarrho-rheumatic cases. Professor Beer mentions that it originates in a phlyctenula, but I have never had an opportunity of seeing any appearance of this kind. If the case continues to be neglected, or if it be mistreated, this ulcer ceases to be superficial; the substance of the cornea is more deeply attacked, and an opaque leucoma will be the result.

Onyx, or effusion of pus between the lamellæ of the cornea, is the most alarming of all the symptoms of this ophthalmia. It generally commences at the lower edge of the cornea, in shape like the white spot at the root of the nails, convex on its upper edge, gradually increasing, mounting upwards, separating more and more the lamellæ between which it is effused, and greatly adding to the sufferings of the patient. It reaches not unfrequently to such a height as to implicate more than half of the cornea. The pus of an onyx in catarrho-rheumatic ophthalmia is very rarely absorbed. The cornea becomes ulcerated over the centre of the onyx; the pus is evacuated; the ulcer but too often penetrates through the posterior lamellæ of the cornea; the aqueous humour escapes; the iris falls forward into contact with the ulcerated cornea; in nine cases out of ten these parts adhere together, and the result is partial or total staphyloma." 414.

As the onyx advances, lymph is commonly effused into the pupil, which becomes sluggish, then hazy, contracts, and may at last be obliterated. Sometimes the onyx is accompanied by hypopium, or effusion of pus into the anterior chamber. At other times the onyx bursts first into the anterior chamber, false hypopium is produced, and ultimately the cornea gives way. If even the onyx is absorbed, albugo remains for a considerable time, but gradually diminishes, and at last may almost disappear. If the onyx is dispersed by the cornea giving way, leucoma is the result, and never entirely disappears. Unless the iris and cornea have become partially or generally adherent staphyloma cannot exist; it is generally at the inferior half of the cornea, because it is commonly the consequence of onyx, which mostly takes place at the lower edge of the cornea. The pulse is generally quick and sharp, and catarrh sometimes adds to the febrile symptoms. The rheumatic symptoms generally yield to treatment first, the catarrhal remaining for some days afterwards. Sometimes our author has observed the reverse.

*Causes.* It may be traced to the same atmospheric influences, which have already been enumerated, as giving rise to catarrhal and rheumatic ophthalmiæ, and it is mostly prevalent during North-easterly winds. Beer thought it was a mixture of foul air and cold air that occasioned the disease. It is probable that the discharge from an eye thus affected, will, if applied to the conjunctiva of a healthy eye, occasion a puro-mucous conjunctivitis. Beer mentions that catarrho-rheumatic ophthalmia sometimes occurs in children, and still oftener in old persons, along with suppression of urine; nor does he regard it as an accidental coincidence. The disease is much more frequent in the old than in the young or middle-aged.

*Treatment.* Judgment and selection, and not new remedies, are required. Venesection is required, must be active according to the severity of the case and age of the patient, and must be repeated next day, if the symptoms are not greatly relieved. Leeches to the temple are highly useful, especially soon after venesection. Scarification of the palpebral conjunctiva is to be employed when there is any considerable chemosis. Calomel and opium should be administered as in the pure rheumatic ophthalmia. Opiate frictions an hour before the expected paroxysm of circumorbital pain, belladonna, blisters, and purgatives, are all useful; so are sudorifics, and tonics in the chronic stage of the disease. The solution of the argenti nitras speedily relieves the symptoms of conjunctivitis, but has no effect on the sclerotic part of the disease; and our author would be very sorry to trust almost wholly to this remedy. The eye is to be bathed four or five times daily with the tepid murias hydrargyri lotion, one grain to eight ounces, and the red precipitate ointment is to be smeared over the edges of the tarsi at bedtime. In onyx our author does not advise the evacuation of the pus by the lancet, as, where he has done it, partial or general staphyloma has resulted. When he has left the onyx to itself, the case has sometimes recovered astonishingly well. This he attributes to the sorbefacient influence of the calomel over the lymphatic effusion into the pupil which always attends extensive onyx; to the continued use of belladonna; and to the gradual preparation of the cornea by Nature for its giving way, a preparation which must be entirely defeated when we venture to open the onyx with the knife.

### X. SCROFULOUS CORNEITIS.

The cornea is liable to suffer in any of the ophthalmiæ already considered, and in others not yet treated of. But the present disease is essentially different from every other. It appears to be chiefly in the conjunctival layer of the cornea, and the substance immediately beneath that layer.

*Symptoms.* The redness is principally in the sclerotica, and on the surface of the cornea. The sclerotic redness is not usually considerable, of a carmine colour, approaching to purplish, the vessels very minute, and arranged zonularly round the cornea. Not unfrequently there is a reddish ring somewhat elevated, formed around or upon the edge of the cornea, while red vessels, more or less numerous, are traceable over its surface to its centre. In some cases the whole cornea is so covered that it assumes a red colour, and this is called a *pannus.* In chronic cases the visible arteries of the eyeball, derived from the recti muscles, are much dilated.

"The cornea is more or less opaque, and rough. The roughness frequently resembles the dotting which might be produced by touching the surface of the cornea all over with the point of a pin. In other instances, the depressions are somewhat larger, and assume, under the magnifying glass, the appearance of a crowd of minute ulcers. In every case, we find that the surface of the cornea has lost its natural polish; and from this circumstance, even when little opacity is present, the eye appears dull, and vision is indistinct. In some instances, the opacity amounts to haziness only; in others, it consists in a streaked or speckled whiteness, arising from depositions of coagulable lymph, with interstices of clear cornea. Not unfrequently the surface of the cornea becomes completely and almost uniformly white. Here and there we occasionally observe upon it elevated points of a yellowish colour, which never appear to suppurate or ulcerate." 419.

In many cases the cornea is unnaturally convex, or even conical, the aqueous humor superabundant; there is hydrophthalmia. Dilatation of the pupil is not unfrequent, nay, there is often a tendency to amaurosis. In other instances iritis, with consequent adhesions, is set up, but it is often difficult to recognize the state of the iris or pupil through the hazy or speckled cornea. Concentrating the light on the cornea, by a double convex lens, is useful in such cases. There is not usually much intolerance of light, but sometimes this is great, with considerable epiphora. There is little or no pain, except perhaps in the commencement. The pulse is quick, the skin harsh and dry, the patient commonly restless at night. It is most frequent about the age of puberty, in women; often connected with amenorrhœa; and coincident with a peculiar hoarseness of voice. Other strumous symptoms are generally present. The cornea is evidently the chief seat of the disease, and *corneitis* seems the most appropriate name.

*Causes.* They are obscure. Our author has known it arise from exposure during the night to the glare of flambeaux. Cold is probably an operative agent.

*Treatment.* Leeches to the neighbourhood of the eye are very useful, especially in the commencement, or when there is pain or tension in the eye or across the forehead. They ought to be repeated with caution, from time to time. Emetics and purgatives are useful, and the tartar emetic is particularly so, in doses of from the 12th to the 4th of a grain thrice a day, and along with Peruvian bark. Diaphoretics are indicated: the pediluvium and Dover's powder. Mercury, carried so far as to affect the mouth, is of great service.

"It is not to be commenced, however, in general, till the acute symptoms have been removed by depletion of different kinds, and the employment of tartar emetic in small doses. When the mercury begins to act decidedly on the constitution, we generally find that the enlarged vessels on the cornea contract, and the newly deposited matter becomes absorbed. The clearing of the cornea commences around its circumference, the favourable change gradually advancing towards the centre. The best form, in which to administer mercury in this, as in some of the former ophthalmiæ, is calomel with opium. Mercury is peculiarly necessary in those cases which are attended with iritis, and in them ought to be employed from the first." 420.

The sulphate of quina exerts a gradual but very beneficial influence on the disease. Colchicum, sarsaparilla, and elm bark are useful remedies, but inferior to cinchona and quina. In this, as in other things, the practitioner having made choice of a remedy must not rashly and hastily abandon it. Many cases are under treatment for a whole year or even longer, before they perfectly recover. And now for the local means of cure. Warm poppy fomentations, and exposure of the eye to the vapour of hot water and laudanum, give great relief where the presence of light proves irritating. Blisters and issues on the neck, behind the ear, and on the temple, are useful and generally necessary. Stimulants are admissible only after acute inflammation is subdued. Next to vinum opii our author would place the red precipitate salve; about the bulk of a split-pea being introduced daily between the lids and eyeball, and then carefully rubbed upon the surface of the cornea through the medium of the upper lid. From ʒss. to ʒi. of red precipitate, triturated along with an ounce of white sugar into an impalpable powder, and blown into the eye through a quill, is another method of applying the same substance. The lunar caustic solution, and an injection of four grains of sulphate of zinc in an ounce of water, are useful also. It is a good plan to employ one stimulant in the morning and another at bedtime. Belladonna should be used at bed-time when there are symptoms, or even only a suspicion, of inflammation of the iris. Evacuation of the aqueous humour appears to be indicated in those cases in which there is a tendency to hydrophthalmia.

Here we must lay aside our pen, for although the inflammations of the iris, choroid, retina, lens and its capsule, and hyaloid membrane, which our author treats of next, have no natural disjunction from the subjects already discussed, yet something like a break does really exist, and of that our limits compel us to take advantage. If we have very closely adhered to the plan of analysis, and avoided criticism, it is not altogether that we had no observations to offer, but because we wished to put our readers in possession of Mr. Mackenzie's practice and opinions, rather than those of ourselves or others. Laws are, or should be, for the many, and so are periodical publications. The literary few may be tired of any thing save keen remark and tranchant criticism. But of what utility are such to the million? The country surgeon, unable to purchase the expensive books which are daily poured from the press, and yet unwilling to remain behind more fortunate compeers, in the knowledge of the day, naturally resorts for useful information to those publications which purport to be its vehicles, we mean the medical journals. It may be more amusing to peruse a sharp cutting little article, but we venture to doubt whether learning of this kind will enable him to get a patient or to keep one. We have always endeavoured to embody in this journal the greatest possible quantity of useful facts in a very moderate space. Much of what is already known must necessarily be repeated, for if novelty only was selected, we could not fill twenty pages in a year, and if so filled, they would probably not be worth reading. We write, we repeat, for the body of the profession, and if we are the means of conveying practical information to those who would otherwise remain without it, the true objects of journal and journalist are then attained.

It is hardly necessary to pronounce a critical judgment on Mr. Mackenzie's work. We have no doubt that it will become the class-book of all who take an interest in the diseases of the eye, for it forms almost a library in itself. It is in fact too voluminous, and many points are treated of in a prolix and methodical manner which scarcely required notice. The ophthalmic surgeon will hardly wade through the whole book, nor was it intended that he should do so. The work is for the student, young or old, and to the student we recommed it. But let not our meaning be misunderstood. We do not recommend this Treatise to the student only, for we believe that many men who are conversant with the department of surgery of which it treats, may very beneficially scan its pages. On the whole then we can pronounce a very favourable opinion of the book, and we think we can assure Mr. Mackenzie of the author's highest heaven, an extensive sale.

